# Identification of Ubiquinones in Honey: A New View on Their Potential Contribution to Honey’s Antioxidant State

**DOI:** 10.3390/molecules23123067

**Published:** 2018-11-23

**Authors:** Katrina Brudzynski, Liset Maldonado-Alvarez

**Affiliations:** 1 Department Drug Discovery and Development, Bee-Biomedicals Inc., St. Catharines, ON L2S 3A1, Canada; 2 Department of Biochemistry, McMaster University, Hamilton, ON L8S 4L8, Canada; maldonl@mcmaster.ca

**Keywords:** ubiquinone, honey, LC-ESI-MS, UPHLC-ESI-MS/MS, protein-polyphenol complexes

## Abstract

Honey is composed of macromolecules arranged into multicomponent colloidal particles dispersed in a supersaturated sugar solution. The core part of colloidal particles in honey is made up of high-molecular weight protein-polyphenol complexes. We designed a multi-step extraction process to gain better insight into the phenolic compounds strongly bound to proteins in honey. Honeys were sequentially extracted by solvents of reduced polarities and the extraction process was monitored by LC-ESI-MS/MS. Unexpectedly, the results revealed ubiquinone-like compounds that partitioned to both, soluble supernatants and protein-bound insoluble residues from which they were released after the pronase-digestion of proteins. The accurate mass measurement and MS/MS fragmentation patterns using UPHLC-MS/MS coupled to quadrupole orbitrap confirmed their identification as ubiquinones. Distribution of ubiquinone-bound proteins was further investigated by the fractionation of honey protein-polyphenol complexes by size-exclusion chromatography followed by LC-ESI-MS analysis. Mass spectra revealed the presence of ubiquinones (UQs) in fractions of high polyphenol to protein ratio. The dominant mass peaks observed in these fractions were identified as UQ-3, UQ-5, and UQ-7. Since the quinone group of UQs is involved in redox reaction, we discuss the possibility that UQs may contribute to the antioxidant/proxidant activity of these complexes.

## 1. Introduction

Protein-polyphenol complexes are an integral part of honey colloids [1,2,3]. Honey proteins, through the interaction with other proteins and polyphenols, are able to form high molecular weight complexes and aggregates. The driving force in the protein-polyphenol complexation is a hydrophobic force/effect which is responsible for the formation of colloidal particle. Due to the hydrophobic effect, the hydrophilic proteins exposed to the outside of particle interact with aqueous sugar solution while burying nonpolar, hydrophobic molecules, such as polyphenols and other terpenoids with ring structures, inside the colloid [4,5]. Assembly into transient or irreversible protein-polyphenol complexes depends on whether the interaction between these molecules leads to formation non-covalent or covalent bonds. The type of the bond, in turn, is determined by the polyphenol basic structure, the degree of hydroxylation, glycosylation on one hand and acylation, conjugation with other phenolics, and polymerization on the other hand, respectively [2,5,6,7].

Relatively little is known about the chemical nature of honey phenolics that are covalently bound to proteins. The aim of this study was to identify phenolic compounds able to form strong complexes with honey proteins. To get some insight into their structure, we employed liquid–liquid sequential extraction of honey with solvents of reduced polarities. Polar solvents such as are ethanol, methanol, acetone, and ethyl acetate in different proportion to water are commonly used to extract non-covalently bound phenolics [8,9,10]. Nonpolar phenolic polymers can be extracted with less polar solvents like acetone, hexane, chloroform, or dichloromethane [8]. In contrast, the covalent binding to proteins might render phenolic compounds non-extractable by conventional solvents. In such a case, enzymatic or alkaline hydrolysis of proteins is required to release phenolics that remained bound after liquid–liquid extraction. With this in mind, we developed a simple method of extraction of phenolics from protein-polyphenol complexes in honey. The phenolic compositions of the free and bound fractions were analyzed by LC-ESI-MS methods. However, unexpectedly, the extraction procedure provided unknown isoprenoid quinones that partitioned into soluble (supernatants) and particulate, protein-bound fractions. Mass spectra and exact mass measurements identified these compounds as ubiquinone UQ-3, UQ-5 and UQ-7.

## 2. Results and Discussion

### 2.1. Extraction and Separation of Ubiquinone-Like Compounds

**Figure 1:**
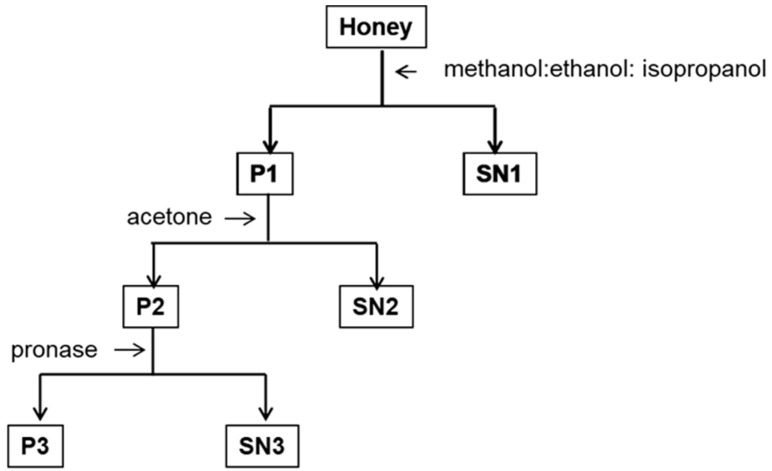
A general outline of liquid–liquid extraction of honey.

Honey high molecular complexes were precipitated using a mixture of methanol: ethanol: isopropanol (1:1:1), followed by acetone extraction and finally, followed by pronase digestion of the water-insoluble pellet. The use of solvents of reduced polarity aimed to increase the yield of extraction of phenolic compounds of different chain length and hydrophobicity. Obtained supernatants, SN1, SN2, and SN3 were lyophilized, dissolved in methanol and cleanup by solid-phase extraction. The composition of the extracted compounds was analyzed by LC-ESI-MS (Figure 1).

**Figure 2:**
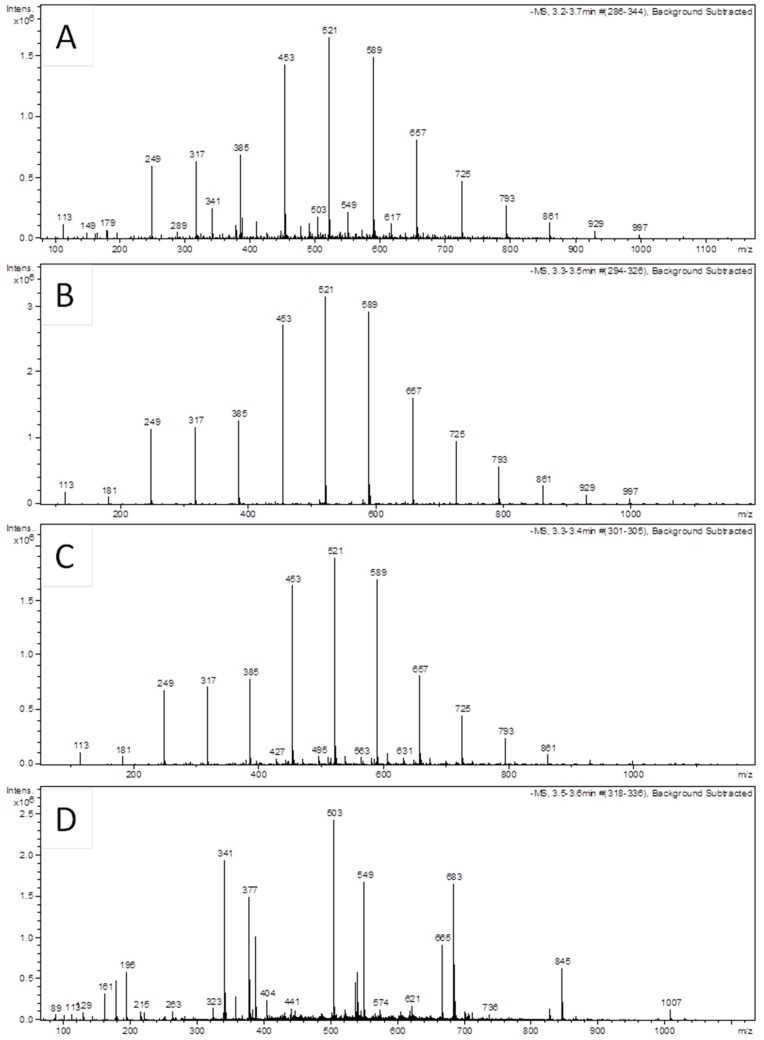
Mass spectra of ubiquinone-like isoprenoid compounds extracted from honey with different solvents and after protein degradation with pronase. (**A**) A full scan mass spectrum of extractives in SN1 removed by a mixture of methanol: ethanol: isopropanol, (**B**) extractives in SN2 after acetone extractions and (**C**) extractives in SN3 after pronase digestion. (**D**) Mass spectrum of extractives present in the supernatant after precipitation of honey with 80% ethanol only.

Unexpectedly, the extraction of honey with a mixture of three alcohols and then with acetone removed substantial amounts of unknown phenolic compounds that gave a sequential pattern of mass peaks differing by 68 Da, corresponding to isoprenyl group (Figure 2). Under our LC-ESI-MS conditions (Materials and Methods), these compounds were eluted at RT 3.3–3.4 min out of 25 min total time. Twelve ion peaks observed in the full scan mass spectra, conducted in negative ionization mode, ranged from [M − H]^−^ *m*/*z* 181 to *m*/*z* 997 (Figure 2, Table 1). Mass spectra of SN 1 and 2 were virtually indistinguishable from one another, indicating that both solvents led to solubilization and removal of lipid-like compounds with polyprenyl chain (Figure 2). In contrast, under the same LC-ESI-MS conditions, the supernatant obtained from honey precipitation with 80% alcohol only, gave mass spectrum at this RT (3.3–3.5 min) devoid of the peculiar polymeric compound (Figure 2D). The characteristic fragmentation pattern where the main mass ions sequentially differed by an isoprene unit of 68 Da is representative of isoprenoid quinones such as menaquinones and ubiquinones. Upon electron impact, both isoprenoid quinones produce product ions consistent with the formula [M − 69] − (n × 68) [11]. By comparing the mass peaks obtained in this study with the PubChem data on isoprenoid quinones, we found that all mass ions shown on our mass spectra (Figure 2), starting from [M − H]^−^ *m*/*z* 181 up to *m*/*z* 997, were perfectly matched with that of ubiquinones (Table 1).

### 2.2. UHPLC-ESI-MS/MS Analysis and Identification of Ubiquinones

To definitively identify these polyprenyl compounds as ubiquinones, the acetone extracts of honey H177, H220 and H208, were analyzed using high resolution UPHLC-ESI-MS/MS, in positive ionization mode, coupled to quadrupole orbitrap. From the full scan mass spectra that generated distinct peaks on the total ion chromatogram (Figure 2), we selected four peaks and used them as a quarry for the extracted ion chromatograms (EICs). The selected ions for the EIC corresponded to the following [M + H]^+^: *m*/*z* 386, *m*/*z* 523, *m*/*z* 659 and *m*/*z* 794. Knowing that the ubiquinone chemical structure consists of 2, 3-dimetoxy-5-methyl-6-polyprenyl-1, 4-benzoquinone where a polyisoprene chain can range from 1 to 12 isoprene units, we presented calculated, theoretical masses of UQ 1 to 12 (Table 1). For the UQ identification, we combined the high resolutions EIC mass spectra with the exact mass measurements by orbitrap to get mass ions accuracy within 5 ppm error of expected theoretical formula. This strategy enabled us to positively identify the following peaks; *m*/*z* 386.246 as UQ-3, *m*/*z* 523.3782 as UQ-5, *m*/*z* 659.5829 as UQ-7 and *m*/*z* 794.5476 as UQ-9 in honeys H208, H220 and H177 (Table 1). All these UQs generated a base peak at *m*/*z* 198.2090 in MS/MS spectra which is considered a diagnostic for ubiquinones (Table 1).

### 2.3. Partitioning of Ubiquinones between Free and Protein-Bound Fractions

**Figure 3:**
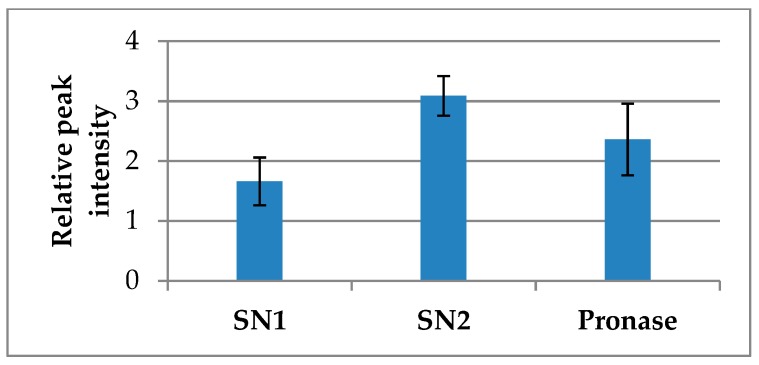
Partitioning of ubiquinones between free (supernatants, SN1 and SN2) and protein-bound fractions released by protease digestion (Pronase).

While ubiquinones were easily extracted from honey with the mixture of three alcohols and acetone, the substantial amount of UQ was freed only after pronase digestion of the insoluble pellet left after centrifugation (Figure 3). Pronase digestion was carried out after the extensive wash of the pellet with acetone, removal of acetone under a stream of nitrogen gas and re-suspension of the pellet residue in a pronase buffer. After protein hydrolysis, undigested residue was removed by centrifugation and the supernatant was cleaned-up by solid-phase extraction on C_18_ cartridges, eluted with 100% methanol, concentrated by lyophilization and finally analyzed by LC-ESI-MS. The dominant peak *m*/*z* 521 observed in all SNs was used to assess the abundance/yield of ubiquinones obtained after each solvent extraction and by pronase digestion in three honeys. Comparison of the mean relative intensity of peak *m*/*z* 521 in each supernatant showed that the significant portion of UQ remained with the protein complexes after extraction with organic solvents (Figure 3).

The partitioning of UQs suggested the existence of two groups of UQ that bound to proteins with different strength. Considering ubiquinones structure consisting of a hydrophilic head group and hydrophobic isoprenoid side chain, it could be inferred that this molecule binds protein in non-covalent way via both hydrogen bonds and hydrophobic interactions. Such transient, reversible associations can be easily disrupted by polar solvents and allow UQs partitioning into supernatants SN1 and SN2.

In contrast, quinone groups of ubiquinones could form covalent adducts with proteins via interaction with protein ε-NH2 or SH groups. We have previously revealed quinone-protein conjugates in SDS-PAGE gels by using nitro blue tetrazolium stain [12], a stain that has been developed for the specific detection of quinone-adducted proteins [13]. The quinone-protein specific staining was increasingly visible on SDS-PAGE among “polyphenol-type” protein-polyphenol complexes, which we isolated from honeys using size-exclusion chromatography [12].

We therefore asked whether “polyphenol-type” protein-polyphenol complexes indeed contain UQ.

### 2.4. Extraction of Ubiquinones from Polyphenol-Protein Complexes

We have previously reported that honey protein-polyphenol complexes could be crudely resolved by size-exclusion chromatography (SEC) into two groups differing in molecular size, protein to polyphenol ratio and the antioxidant capacity [12]. Although the molecular size range is difficult to determine accurately from SEC, we estimated the size of these two groups of high molecular weight complexes to be within 230–180 kDa and 110–85 kDa range. Based on the protein and polyphenol concentrations and their ratio, the first group was found to be enriched in protein (“protein-type” complexes) while lower molecular size complexes (110–85 kDa) were enriched in polyphenols (“polyphenol-type” complexes) [12].

**Figure 4:**
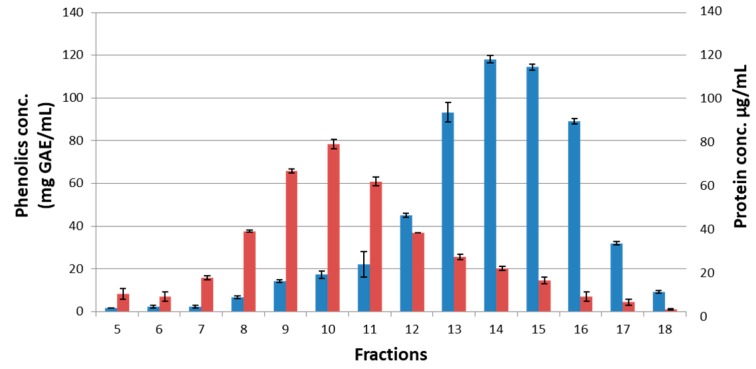
The total phenolic and protein contents in fractions obtained from size-exclusion chromatography of honey H177 on Sepharose 4B.

To determine whether ubiquinones could be found among the “polyphenol-type” complexes, honeys H208, H177 and H220 were chromatographed on Sepharose 4B to separate fractions differing in the protein to polyphenol ratio. Each fraction was analyzed for the protein and polyphenol contents using Bradford protein assay and Folin-Ciocalteu method, respectively. Fractions 8–12 showed higher protein concentrations than fractions F13 to F17 (two-tailed t-test, t(8) = 4.6. *p* < 0.002), while fractions F13 to F17 showed higher phenolic concentrations than fractions F8-F12 (t(8) = 4.07, *p* < 0.003). Fractions F15 to F17 rich in polyphenols were selected for the LC-ESI-MS analysis (Figure 4).

**Figure 5:**
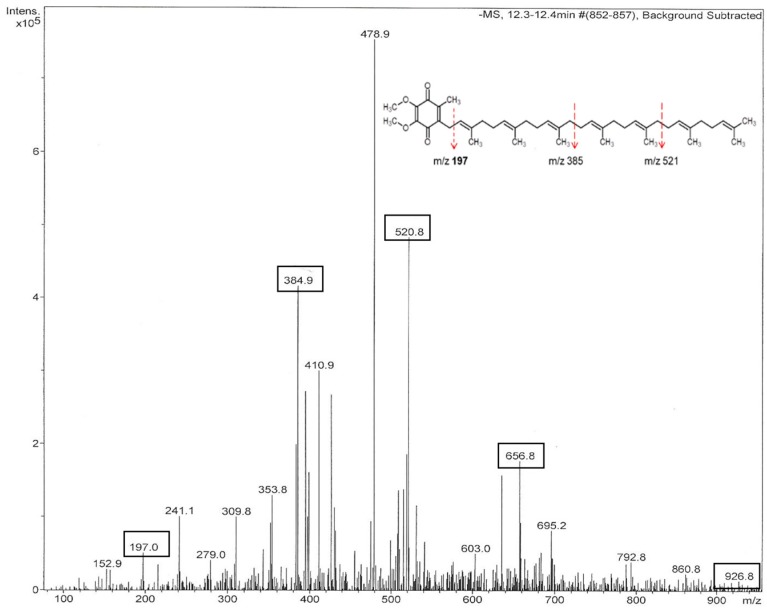
Mass spectrum of fraction F15 of buckwheat honey H177 conducted in the negative ionization mode. The insert provides a general structure of ubiquinone UQ-7, *m*/*z* 656.6 and its fragmentation patterns. The product ion spectrum includes *m*/*z* 520.8, *m*/*z* 384.9 and *m*/*z* 197.0.

Mass spectra of the fractions F15 to F17 obtained in the negative ionization mode showed strong signals of the [M − H]^−^ at *m*/*z* 384.9, *m*/*z* 520.8, *m*/*z* 656.8 and occasionally, *m*/*z* 928.9 (Figure 5). These peaks were consistently eluted at a retention time 12.1–12.4 min (Table 2). Interestingly, the peak mass ions differed by two mass units of isoprene suggesting that they might be formed by cleavage of the ubiquinone with the longer polyprenyl side chain, such as that of *m*/*z* 928.9.

The high intensity of the *m*/*z* 384.9 in mass spectra of the tested fractions might be related to degree of polymerization of polyprenyl side chain rather than to the relative abundance of this compound in the fractions, since the relative intensity of the peak is inversely correlated with the number of isoprene units in polyprenyl side-chain. The product ion at *m*/*z* 197.1 represents benzoquinone nucleus, 2,3-dimetoxy- 5,6-dimethyl- 1,4 benzoquinone (Figure 5, insert) that is produced from the successive reduction of terminal isoprene units of polyprenyl chain. The fragment ion *m*/*z* 197.1, a peak signifying ubiquinones, was consistently observed in mass spectra of all tested fractions (Figure 5 and Table 2). Mass spectra of fractions F15 to F17 indicate the presence of ubiquinones UQ-3, UQ-5, UQ-7 and UQ-9 in the polyphenol-enriched protein-polyphenol complexes in honey.

Our findings indicated that ubiquinones have emerged as significant components of protein bound-compounds in honey. We therefore, raised the question as to their functional significance. We perceive at least two possible outcomes based on known facts on the function of UQ in living organisms:

In prokaryotic and eukaryotic cells, ubiquinones are indispensable components of the large respiratory protein complexes spanning the inner mitochondrial membrane (or plasma membranes of bacteria) responsible for energy production. This involves the transfer of electrons produced in the redox reaction of quinone group of ubiquinones and its coupling with the pumping of H^+^ across the membrane. The latter process generates a proton gradient (proton motive force) which is used by ATP synthase to synthesize ATP [14,15]. Thus, these large respiratory protein-ubiquinone complexes comprise an operational bioenergetic system producing energy via quinone’s redox ability. Would a similar bioenergetic phenomenon be observed in honey? In other words, would polyphenol-type protein-polyphenol complexes containing ubiquinones continue to be involved in electron transfer in honey?

Secondly, we have shown previously that polyphenol type complexes possessed higher antioxidant activity, measured by ORAC method, than protein-type complexes [16]. The ORAC values of fractions rich in polyphenols (F13 to F15) was significantly higher and showed a strong correlation with the total phenolic content, R^2^ = 0.860, *p* < 0.0001. The presence of the redox-active ubiquinones in the former type complexes could imply ubiquinone’s contribution to this antioxidant activity. In support, the literature evidence indicate that ubiquinol, the reduced form of ubiquinone, has the ability to scavenge superoxide and lipid peroxyl radicals in several in vitro systems and therefore has ability to serve as an antioxidant [15,17,18].

Our findings prompt us toward new research directions into the role ubiquinones and menaquinones in honey and their potential to influence the honey’s antioxidant/ proxidant state.

## 3. Materials and Methods

### 3.1. Honeys

Honeys were donated by Canadian beekeepers and included apiary (raw) samples (Charlie-Bee Honey, Beamsville, ON, Canada; Honey Q Corp. Sunderland, ON, Canada; Dutchman’s Gold Honey, Carlisle, ON, Canada). Three honeys used in this study, H177, H208, and H220 originated from buckwheat (*Fagopyrum esculentum*). Honeys H177, H208, and H220 were collected during the 2010, 2013, and 2016 seasons. Upon their arrival to the laboratory, honeys were given ID numbers, aliquoted to the separate, dark-color jars and stored at −20 °C. Each honey was extensively characterized as to the color, moisture, water content, Brix, and specific gravity (Table 3) as recommended by the Harmonized Methods of the International Honey Commission [19].

### 3.2. Sample Preparation

Honey samples were diluted to 50% (*w*/*v*) with warm (45–50 °C) Milli-Q water and the resulting solution was filtered using glass microfiber Centrex units (Schleicher & Schuell Inc., Keene, NH, USA) followed by filtration through a 0.45 μm syringe membrane filter (VWR, Radnor, PA, USA).

### 3.3. Extraction Procedures

The 50% honey aqueous solution (500 µL) was extracted with the 500 uL mixture of three alcohols, 80% ethanol, 80% methanol and 70% isopropanol (1:1:1), incubated for 30 min at room temperature, in the dark, and centrifuged at 6400 rpm/10 min. to collect a supernatant (SN1) and the pellet. The pellet was suspended in 200 µL of Milli-Q water and extracted with 800 µL of ice-cold acetone overnight at 4 °C. After centirfugation, the precipitate, separated from supernatant (SN2), was rinsed 3 times with 200 µL of acetone followed by centrifugation. The rinsed pellet was evaporated to dryness under a stream of nitrogen gas to remove acetone residue. The dried precipitate was suspended in 900 µL of Milli-Q water by vortexing and mixed with 100 µL of pronase (5 mg/mL, *w*/*v*) in 10 mM sodium acetate buffer containing 5 mM CaCl_2_, pH 7.5. The sample was incubated for 24 h at 37 °C in the dark. The reaction mixture was centrifuged and the supernatant was then applied to SPE column and eluted with 100% methanol (SN3, Pronase). The supernatants SN1, SN2, and SN3 were lyophilized. Lyophilization was conducted using a benchtop freeze-dryer system at −50 °C and vacuum at 380 mBar (Thermo Savant Micro Moduly, Marshall Sci., Hampton, NH, USA). Samples were reconstituted in 20 µL methanol. 5 µL of the sample was injected and analyzed by LC-ESI-MS (Bruker HCT Ultra LC/MS, Billerica, MA, USA).

### 3.4. Solid Phase Extraction (SPE)

Solid phase extraction was used in this study for a purpose to remove interfering substances and impurities from samples before the determination of ubiquinones in supernatants by LC-ESI-MS (UPHLC-MS/MS) or the total phenolic content by the Folin–Ciocalteau method. Waters Oasis HLB 3cc Extraction Cartridges (Oasis HLB, Waters Corp, Milford, MA USA) were used according to the manufacturer manual.

### 3.5. LC-ESI-MS Analysis

LC-ESI-MS analysis was performed with a Bruker HCT Ultra LC/MS instrument using a ZORBAX Eclipse XDB-C18, 4.6 × 50 mm column (Agilent). The injection volume was 5 μL. The mobile phase consisted of solvent A (0.1% formic acid in water) and solvent B (0.1% formic acid in methanol). Chromatographic separation was 25-min. The gradient program was as followed: 0–3 min, 22% B; 10 min, 100% B; 12 min 100% B; 13 min, 22% B; 16 min, 22% B. ESI was run with negative ion polarity with a capillarity exit voltage of −128.5 Volts. The elution was monitored at 254 nm. The method of ionization was electrospray ionization (ESI). The LC-ESI-MS was operated on a negative ion mode with a mass range of 100–1000 *m*/*z*.

### 3.6. Ultra-Performance Liquid Chromatography (UPLC)

Fractions from size-exclusion chromatography purified by SPE were subjected to chromatographic separation using an ACQUITY UPLC (Waters, Milford, MA, USA) containing a Waters Acquity BEH C18 column (150 mm × 2.1 mm, 1.8 μm) with a flow rate of 0.5 mL/min at 30 °C. The injection volume was 5 μL. The mobile phase consisted of solvent A (0.1% formic acid in water) and solvent B (0.1% formic acid in acetonitrile). Chromatographic separation was 6 min. The gradient was 0 min, 8% B; 1 min, 8% B; 4.3 min, 25% B; 5 min, 8% B; 6 min, 8% B. The method of ionization was electrospray ionization (ESI). The UPLC-ESI-MS was operated on a positive ion mode with a mass range of 100–1000 *m*/*z*. The cone voltage was set to 30 V, the source temperature at 147 °C; the desolvation gas flow was 483 L/h at a temperature of 300 °C. The detection was diode-array detection that was set up at 248 nm to optimize the detection of benzoquinones. The data acquisition and processing was obtained using Thermo Xcalibur 2.2 software (Thermo Fisher Scientific, Waltham, MA, USA).

### 3.7. The Exact Mass Measurement

The exact mass measurements were done using Q-Exactive mass spectrometer. The LC-MS platform consisted of a Dionex Ultimate 3000 UHPLC system and a Q-Exactive mass spectrometer equipped with a HESI II source (Thermo Scientific). Control of the system and data handling was performed using Thermo XCalibur 2.2 software and Chromeleon 7.2 software. Separation by liquid chromatography was conducted on an Acquity BEH C18 column (50 mm × 2.1 mm, 1.7 μm particle size). The pump was run at a flow rate of 200 μL/min. Solvent A was water containing 0.1% formic acid; solvent B was acetonitrile containing 0.1% formic acid. The gradient was 0 min, 8% B; 6 min, 95% B; 8 min, 95% B; 9 min, 8% B; 15 min, 8% B. Autosampler temperature was maintained at 10 °C and injection volume was 15 μL. Data collection was done in positive ionization mode with MS1 scan range *m*/*z* 100–1000, resolution 70,000, AGC target of 3e6 and a maximum injection time of 200 ms, MS2 data was collected using a TOP5 method, 1 *m*/*z* isolation window, 20, 30 stepped NCE, 17,500 resolution, AGC target 1e5 and a maximum injection time of 50 ms.

### 3.8. Size-Exclusion Chromatography (SEC)

A Sepharose 4B (Sigma-Aldrich, St. Louis, MO, USA) column (24 cm × 1.6 cm) was equilibrated with distilled water at 1 mL/min prior to use. A 50% (*w*/*v*) honey solution in 0.15 M NaCl was centrifuged at 13,000 rpm for 15 min and one milliliter of the SN was loaded onto the column. The fractions (3 mls) were eluted with distilled water (1 mL/min) and monitored at 280 nm. A standard curve for molecular weight determination was generated using a protein kit (Gel Filtration HMW Calibration kit, GE Healthcare, Chicago, IL, USA) containing ferritin (440 kDa), catalase (240 kDa), aldolase (158 kDa) and albumin (66 kDa) with obtained with linearity ranging from R^2^ = 0.92 to 0.98 in several repetitions.

### 3.9. Determination of Protein Concentration

Protein content was determined using the Pierce BCA Protein Assay Kit (Thermo Scientific) in a 96-well format based on Bradford protein assay [20]. The method was performed as described in the manufacturer manual. Protein determination was conducted by addition of 200 μL of Bradford dye reagent (brilliant blue G, 0.1 mg/mL; ethanol, 5% (*v*/*v*); phosphoric acid, 10% (*v*/*v*) and water) to 40 μL of samples, pre-diluted 100 times. The plate was incubated in the dark with shaking for 1.5 h at 37 °C. Following incubation, the absorbance was measured at A_595_nm using the Synergy HT Multi-Detection Microplate Reader (BioTek Instruments, Inc, Winooski, VT, USA). Protein content was calculated from the albumin (2000–62.5 µg/mL) standard curve (R^2^ = 0.99) and was expressed as µg/mL. Measurements were done in triplicate.

### 3.10. Determination of Polyphenol Concentration

The total phenolic content in the SEC fractions was determined with the Folin–Ciocalteu reagent [21]. Briefly, 790 µL of distilled water, 10 µL of high molecular weight solution, and 50 µL of Folin–Ciocalteu reagent were mixed. After exactly 1 min, 150 µL of 20% aqueous sodium carbonate was added, and the mixture was mixed and allowed to stand at room temperature in the dark for 120 min. Detection was achieved at 760 nm. Gallic acid was used as standard (R^2^ = 0.9998). Measurements were done in triplicate.

## 4. Conclusions

In this study we identified two pools of ubiquinones in honey based on how strongly they bound proteins in protein-polyphenol complexes. The ubiquinone group that transiently bound to proteins was readily extracted using polar solvents. The covalently bound UQ required protein proteolytic degradation in order to be released. Ubiquinone distribution among protein-polyphenol complexes separated by size-exclusion chromatography followed by LC-ESI-MS, have shown that ubiquinone UQ-3, 5 and 7 were consistently associated with polyphenol-enriched complexes. Due to the role ubiquinones have in electron transfer and energy production, we discuss the possibility of ubiquinones’ contribution to the antioxidant/proxidant state of honey.

## Figures and Tables

**Table 1:** Major mass ions of ubiquinones detected during solvent extraction of honey.

Compound	Mol. Mass	Detected Mass [M − H]^−^ (*m*/*z*)			Theoretical Mass (Da)	Formula	Accurate Mass [M + H]^+^ (*m*/*z*)
		SN1	SN2	SN3			
**UQ-12**	999.603	997	997	-	998.809	C_69_H_106_O_4_	
**UQ-11**	931.484	929	929	-	930.747	C_64_H_98_O_4_	
**UQ-10**	863.34	861	861	861	862.6839	C_59_H_90_O_4_	
**UQ-9**	794.6	793	793	793	794.621	C_54_H_82_O_4_	794.5476
**UQ-8**	726.6	725	725	725	727.56599	C_49_H_75_O_4_	
**UQ-7**	658.5	657	657	657	658.496	C_44_H_66_O_4_	659.5829
**UQ-6**	590.899	589	589	589	590.432022	C_39_H_58_O_4_	591.4408
**UQ-5**	522.758	521	521	521	522.370911	C_34_H_50_O_4_	523.3782
**UQ-4**	454.308	453	453	453	454.308	C_29_H_42_O_4_	
**UQ-3**	386.532	385	385	385	386.246	C_24_H_34_O_4_	386.246
**UQ-2**	318.413	317	317	317	318.183	C_19_H_26_O_4_	
**UQ-1**	250.29	249	249	249	250.121	C_14_H_18_O_4_	
**UQ-0**	182.17	-	181	181	182.05790	C_9_H_10_O_4_	

**Table 2:** Main mass ions [M − H]^−^ of ubiquinones observed in protein-polyphenol complexes.

UQ Type	Detected [M − H]^−^, *m*/*z*							Diagnostic Fragment
	H208 F15	H208 F16	H220 F16	H220 F17	H177 H15	H177 F16	H229 F15	*m*/*z*
**UQ-3**	384.9	384.9	384.9	384.9	385	385	384.9	197.1
**UQ-5**	520.8	520.8	-	-	521	521	520.9	197.1
**UQ-7**	656.8	656.8	656.8	656.8	657	657	656.8	197.1
**UQ-11**	928.8	-	-	-	929	861	928.9	197.1

**Table 3:** Some physical–chemical properties of tested honeys.

Honey	Color	Source	Water Activity	Refractive Index	Moisture (%)	Brix	Specific Gravity
**H208**	Medium	Buckwheat/golden rod	0.580	1.4900	18.6	79.8	1.4129
**H177**	Dark	buckwheat	0.572	1.4964	16.2	82.3	1.4295
**H220**	Dark	buckwheat	0.601	1.4988	15.8	83.4	1.4370
